# Supplementary material for: Overloaded and Unrestrained: A Qualitative Study with Local Experts Exploring Factors Affecting Child Car Restraint Use in Cape Town, South Africa
Source: Int J Environ Res Public Health. 2020 Jul 10;17(14):4974. doi: 10.3390/ijerph17144974 (PMC7400026; doi:10.3390/ijerph17144974)
Supplement: Supplementary file 1 [file ijerph-17-04974-s001.pdf]

UNIVERSITY OF CAPE TOWN
IYUNIVESITHI YASEKAPA • UNIVERSITEIT VAN KAAPSTAD

Child restraint use in South Africa: interviews with local experts

In-depth interviews

Macro level (e.g. policy makers in health and transport).

Theme of investigation	Questions
Road Safety in Cape Town, South Africa	• Do you think road traffic collisions, injuries and deaths among children are a problem in Cape Town?<br>• How are children transported in the city?<br>• [Probe: is this the same for all socioeconomic groups in the city?]<br>• How do you think we can decrease the incidence of road traffic injuries among children?
Child restraints as an intervention	• Do you think child restraints keep children safe in vehicles?<br>• Should **all** children in a vehicle be restrained?<br>[Probe: what about if there are more than three children in a car. What do you think the parent should do?]<br>• Do you think that getting children into child restraints will make a big difference to the incidence of child road traffic injuries and deaths?<br>• Do you think there are missing elements in the amendment to the National Road Traffic Act passed in October 2014?<br>[Probe: the amendment only covers children up to the age of 3 years to wear child restraints. Please comment.]<br>• How old do you think children should be before they sit in the front seat of a vehicle using an adult seat-belt?
Barriers and facilitators to using child restraints	• Why do you think child restraints are not being used (despite the new law)?<br>• Do you know how much (on average) a SABS approved child restraint costs?<br>• Are there any non-SABS approved child restraints being sold in Cape Town?<br>• What do you think would increase the uptake of child restraints?<br>[Probe: would a borrowing scheme or a give away scheme increase utilization? Comment]<br>• Do you think that parents require more education about the benefits of using a child restraint?<br>• Do you think the private sector has a role to play in child road safety?

	[Probe: If yes, should they be approached to subsidize or donate child restraints?]
Futuristic interventions for road safety	● In your opinion, which other interventions would potentially be successful in reducing child road traffic injuries in the city? [Probe: Do you think targeting interventions directly for children will prove to be beneficial or should road safety in general be improved]<br>● What other policies are needed to keep Cape Town's children safe on the roads?<br>● Do you have any further suggestions?

Meso level (e.g. health care workers, road safety practitioners, researchers).

**Theme of investigation**	**Questions**
Road Safety in Cape Town, South Africa	● Do you think road traffic collisions, injuries and deaths among children are a problem in Cape Town?<br>● How are children transported? [Probe: is this the same for all socioeconomic groups in the city?]<br>● How do you think we can decrease the incidence of road traffic injuries among children?
Child restraints as an intervention	● Do you think that child restraints will save children's lives?<br>● [Probe: what kind of injuries do you think that child restraints will prevent?]<br>● Do you think that child restraints will increase any specific type of injury?<br>● How old do you think children should be before they sit in the front seat of a vehicle using an adult seat-belt?<br>● Should all children in a vehicle be restrained?<br>● [Probe: what about if there are more than three children in a car. What do you think the parent should do?]<br>● Do you think that getting children into child restraints will make a big difference to the incidence of child road traffic injuries and deaths?<br>● Do you think that health workers have a role to play in primary prevention?<br>● [Probe: in some countries doctors and nurses provide brief interventions to parents who bring children in with an injury. Do you think this would be feasible in busy Cape Town health facilities?]<br>●

Barriers and facilitators to using child restraints	• Why do you think child restraints are not being used?<br>• Do you know how much a SABS approved child restraint costs?<br>• Are there any non-SABS approved child restraints being sold in Cape Town?<br>• Do you think that parents require more education about the benefits of using a child restraint?<br>• What other barriers are there to not using child restraints?<br>• Do you think that providing child restraints (either when a mother leaves the maternity centre or at a well-baby clinic) would increase awareness about their usefulness and lead to an increased uptake of their use?<br>• What else do you think could facilitate the uptake of child restraints?
Futuristic interventions for road safety	• In your opinion, which other interventions would potentially be successful in reducing child road traffic injuries in the city?<br>• [Probe: Do you think targeting interventions directly for children will prove to be beneficial or should road safety in general be improved]<br>• Do you have any further suggestions?
